# Impact of Operating Room Efficiencies on Patient Outcomes Following Primary Coronary Artery Bypass Surgery

**DOI:** 10.1093/icvts/ivaf304

**Published:** 2025-12-18

**Authors:** Jay A Patel, Mohamad El Moheb, Raymond Strobel, Anthony V Norman, Alexander M Wisniewski, Matthew P Weber, Steven Young, Andrew M Young, Evan P Rotar, Abdulla Damluji, Michael C Kontos, Alan Speir, Michael Mazzeffi, Jared Beller, Ramesh Singh, Mark Joseph, Clifford E Fonner, Ourania Preventza, Kenan Yount, Nicholas R Teman, Robert Lancey, Mohammed Quader

**Affiliations:** Division of Cardiac Surgery, Pauley Heart Center, Virginia Commonwealth University, Richmond, VA, 23219, United States; Division of Cardiothoracic Surgery, University of Virginia, Charlottesville, VA, 22908, United States; Division of Cardiothoracic Surgery, University of Virginia, Charlottesville, VA, 22908, United States; Division of Cardiothoracic Surgery, University of Virginia, Charlottesville, VA, 22908, United States; Division of Cardiothoracic Surgery, University of Virginia, Charlottesville, VA, 22908, United States; Division of Cardiothoracic Surgery, University of Virginia, Charlottesville, VA, 22908, United States; Division of Cardiothoracic Surgery, University of Virginia, Charlottesville, VA, 22908, United States; Division of Cardiothoracic Surgery, University of Virginia, Charlottesville, VA, 22908, United States; Division of Cardiothoracic Surgery, University of Virginia, Charlottesville, VA, 22908, United States; Inova Heart and Vascular Institute, Falls Church, VA, 22042, United States; Division of Cardiology, Virginia Commonwealth University, Richmond, VA, 23219, United States; Inova Heart and Vascular Institute, Falls Church, VA, 22042, United States; Division of Anesthesiology, University of Virginia, Charlottesville, VA, 22908, United States; Inova Heart and Vascular Institute, Falls Church, VA, 22042, United States; Inova Heart and Vascular Institute, Falls Church, VA, 22042, United States; Division of Cardiothoracic Surgery, Virginia Tech Carilion School of Medicine, Roanoke, VA, 23219, United States; Virginia Cardiac Services Quality Initiative, South Riding, VA, 20152, United States; Division of Cardiothoracic Surgery, University of Virginia, Charlottesville, VA, 22908, United States; Division of Cardiothoracic Surgery, University of Virginia, Charlottesville, VA, 22908, United States; Division of Cardiothoracic Surgery, University of Colorado School of Medicine, Aurora, CO, 80045, United States; Division of Cardiothoracic Surgery, Sentara Cardiothoracic Surgery Specialists, Harrisonburg, VA, 22801, United States; Division of Cardiac Surgery, Pauley Heart Center, Virginia Commonwealth University, Richmond, VA, 23219, United States

**Keywords:** bypass time, coronary artery bypass, operating room efficiency

## Abstract

**Objectives:**

Prolonged cardiopulmonary bypass (CPB) time during coronary artery bypass grafting (CABG) is associated with poor outcomes, however, the association of other operating room (OR) times is less understood. We studied the impact of OR times on outcomes and resource utilization after CABG.

**Methods:**

Patients undergoing isolated primary CABG from a large multicentre regional collaborative were analysed. The impact of risk-adjusted total OR, surgery, non-surgery, CPB, and off-CPB times on morbidity, extubation time, ICU and hospital length of stay (LOS), cost, and mortality, was studied. Multivariable regressions were performed adjusting for STS predicted risk of morbidity or mortality, intraoperative blood transfusion, CPB time, cross-clamp time, presence of a cardiothoracic surgery fellowship program, and year of surgery. Our adjustment accounted for patient and intraoperative factors that contribute to complexity and intraoperative course of surgery. All models incorporated centre as a random effect to account for hospital-level variations.

**Results:**

Among 29 206 patients (mean age 64.8 years, 76% male), median OR, surgery, non-surgery, and CPB times were 308, 235, 72, and 141 minutes, respectively. Longer surgery times were significantly associated with complications, prolonged ventilation, longer ICU and hospital LOS, and mortality. Similarly, increasing non-surgery OR time was significantly associated with worse outcomes, including longer LOS and complications. Each additional 15 minutes in the OR was associated with increased odds of complications, mortality, and cost.

**Conclusions:**

Longer non-surgical OR times are associated with adverse outcomes and increased cost. Improving OR efficiency may contribute to better patient outcomes.

## GRAPHICAL ABSTRACT

**Figure:**
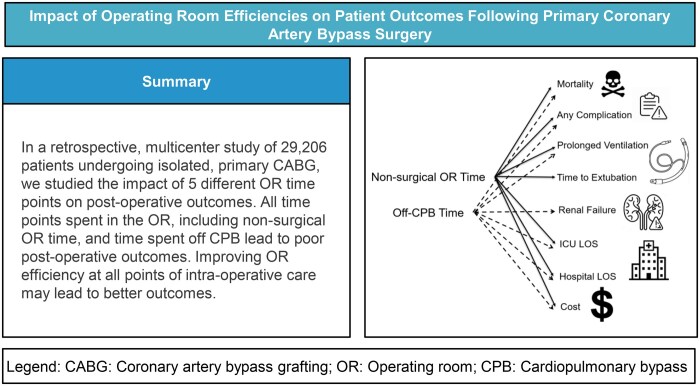

## INTRODUCTION

There are many pre-operative, intraoperative, post-operative, and patient-related variables that impact the outcome of patients who have undergone coronary artery bypass surgery (CABG). Patient risk factors and the impact of cardiopulmonary bypass (CPB) times on outcomes have been well studied. Multiple studies have concluded that prolonged cross-clamp and CPB times are associated with increased mortality and morbidity including pulmonary complications (acute respiratory distress syndrome, ARDS), renal complications, neurologic complications, multiorgan failure, reoperation for bleeding, blood transfusions, and ICU length of stay.^1–7^ With specific operative durations, Madhavan et al associated CPB/graft time over 56 minutes and cumulative CPB time over 180 minutes with worse morbidity and mortality, while Nissinen et al have found that CPB time over 240 minutes and cross-clamp time over 150 minutes are higher risk of developing adverse events independent of complexity of surgery and operative risk.^1^^,^^3^

Several studies have looked at the impact of total operative time on patient outcomes. Cheng et al performed a meta-analysis of prolonged operating room (OR) times across multiple non-cardiac surgical specialties and have found that increased operative duration leads to increased complications with doubling of the odds ratio (OR) with operative duration more than 2 h (OR 1.99, *P* < .001) and an increase in odds ratio with increasing increments of operating room time (OR 1.21 for every 60-minute increase, *P* < .001).^8^ Prolonged surgery time increases the risk of surgical site infection (SSI) by 13%, 17%, and 37% for every 15, 30, and 60 minutes of surgery, respectively, that patients with SSI had, on average, a 30 minutes longer surgery time, and that there is a 14% increase in complications with every 30 minutes of operative time.^8^^,^^9^ Studies looking at the impact of non-operative times on patient outcomes in cardiac surgery are very limited. Chu et al showed that in CABG patients, total surgery time, defined as time from incision to when patient leaves the OR, did not affect short-term survival, but independently predicted ICU LOS.^10^ Mork et al showed an “intervention time” greater than 300 minutes is significantly related to primary bloodstream infection.^4^

Interestingly, the impact of non-CPB OR times, such as the total amount of time the patient spends in the OR (OR entry to exit), total amount of surgery time (skin incision to skin closure time), total time in the OR not spent in surgery (all time in OR excluding skin to skin time), and surgical time not spent on CPB, have not been well studied. Our hypothesis is that longer non-CPB and non-surgery times can negatively impact post-operative patient outcomes. This retrospective study aims to determine the association between OR time and patient outcomes and resource utilization after elective and urgent CABG.

## PATIENTS AND METHODS

### Study population

The study population comprised all adult patients (age >18 years) who underwent isolated, primary CABG between January 1, 2011, and December 31, 2023. Data were sourced from the Virginia Cardiac Services Quality Initiative (VCSQI), a prospectively maintained database (ARMUS Corporation, San Mateo, CA, United States) that captures Society of Thoracic Surgeons (STS) demographic, pre-operative, clinical, and 30-day outcomes data from 17 participating non-governmental institutions. IRB approval was not required with this database. Exclusion criteria included emergent CABG, off-pump CABG, previous sternotomy, and outlying OR times (<1st or >99th percentile).

### Study groups and outcomes

**Figure 1.**
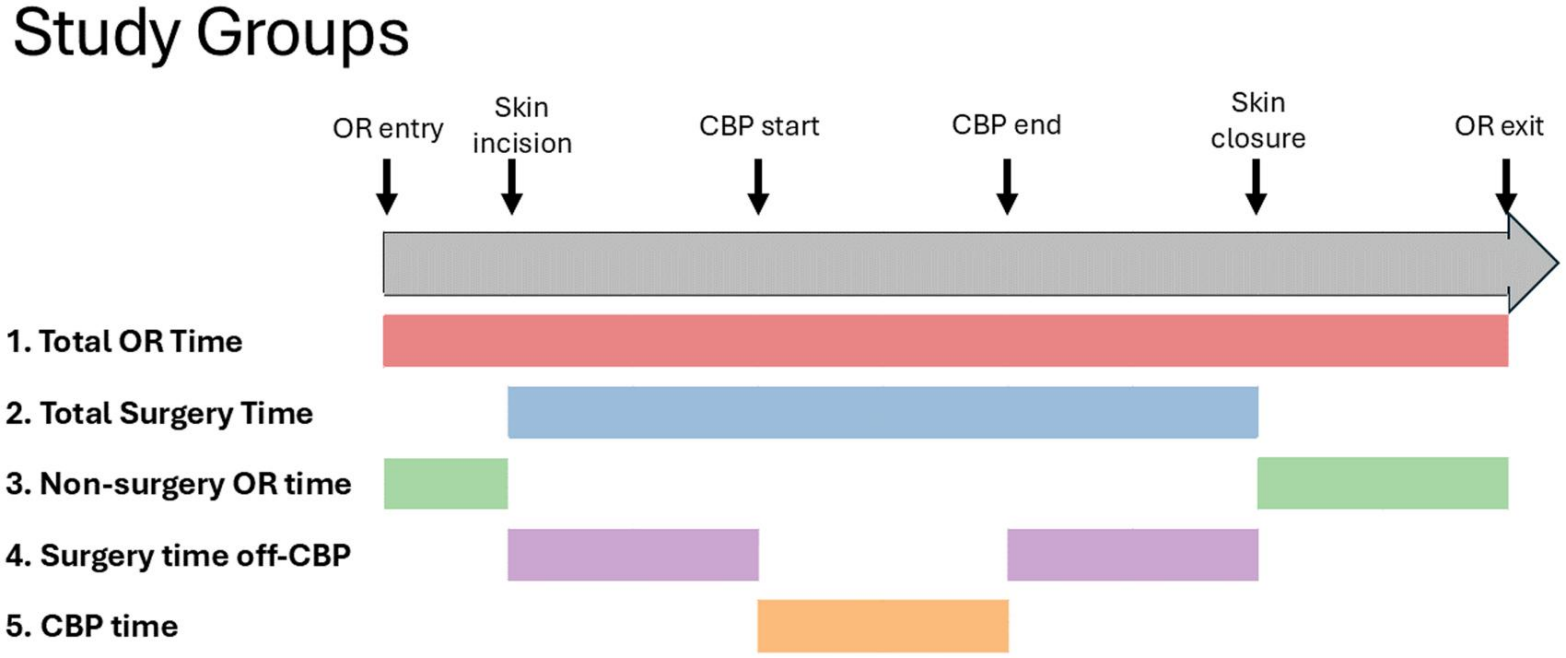
Breakdown of Operating Room Times.

The study examined various measures of OR time, as illustrated in **Figure 1**. Total OR time was subdivided into surgery time (skin incision to skin closure) and non-surgery time (patient entry into the OR to skin incision, and skin closure to patient’s exit out of the OR). Surgery time includes the conduct of the entire operation from skin incision, conduit harvest, initiating and weaning from bypass, performing the bypasses, and hemostasis/closure. Non-surgery time includes pre-incision delays such as anaesthesia interventions, prepping the patient, and post-operative preparations for transfer and transfer holds. Additionally, time on and off CPB (total surgery time excluding the time spent on bypass) was analysed. The relationship between each OR time measure and resource utilization was studied, with time included as a continuous variable measured in 15-minute increments. Two programmes within the VCSQI have training programs in cardiothoracic surgery.

Primary outcomes include all-cause 30-day morbidity, which include sepsis, prolonged ventilation, renal failure (defined as Stage 3 acute kidney injury using the Kidney Disease: Improving Global Outcomes (KDIGO) definition), bleeding, pneumonia, and stroke, as defined by the STS, and 30-day mortality. Secondary outcomes include time to extubation, ICU LOS, hospital LOS, year-adjusted total cost, and impact of teaching status on OR time. Additionally, the impact of increasing 15-minute intervals on morbidity, mortality, and cost was studied. Cost corresponds to the total hospitalization cost, reflecting the total dollar amount billed for the entire hospital stay, encompassing all associated services and treatments.

### Statistical analysis

Continuous variables were reported as means with SD, and categorical variables as frequencies with percentages. Comparisons between continuous variables utilized independent *t*-tests, while categorical variables were compared using *χ*^2^ tests. Multivariable logistic regression models were constructed for categorical outcomes, and multivariable linear regression models for continuous outcomes. To account for hospital-level variations, all models incorporated hospital as a random effect and were adjusted for STS PROMM (which adjusts for number of diseased vessels), intraoperative blood transfusion, CPB time (except when examining the association between time on/off CPB with post-operative outcomes), cross-clamp time, presence of a cardiothoracic surgery fellowship program, and year of surgery. The STS PROMM is a very validated tool for adjustment and captures risk in patients of varying operative risk. The marginal predicted probability of each categorical outcome per amount of time spent in the OR was calculated. For continuous outcomes, the marginal outcome was directly calculated against time spent in the OR. Marginal plots were conducted to visualize these relationships.

The rate of missing data for independent variables was less than 5%. These observations were excluded from the multivariable analysis, and a complete case analysis was performed. All tests were 2-sided, with a *P*-value <.05 considered statistically significant. Data analyses were conducted using R version 4.3.2 (The R Foundation for Statistical Computing) and the following packages: tidymodels, haven, lme4, lmerTest, margins, stringr, ggeffects.

### Secondary analysis

A secondary analysis was performed to evaluate the impact of cardiothoracic surgery training on OR times. Linear regression models were constructed with each of the OR times measured as continuous variables. To account for hospital-level variations, the data was adjusted for STS PROMM, blood transfusion, CPB and cross-clamp time, presence of cardiothoracic surgery fellowship, and year of surgery.

## RESULTS

A total of 29 206 patients were included in the study. The mean age of patients was 64.8 years (SD: 10) and 76% were males. The characteristics of the patient population are presented in **Table S1**. Total OR time ranged from 198 to 513 minutes, surgery time ranged from 132 to 415 minutes, non-surgery time ranged from 41 to 155 minutes, and off-CPB time ranged from 145 to 363 minutes (**Table S2**). There was a weak correlation between surgical and non-surgical time. The results of each studied primary and secondary outcome are described below.

### Total or time

**Figure 2.**
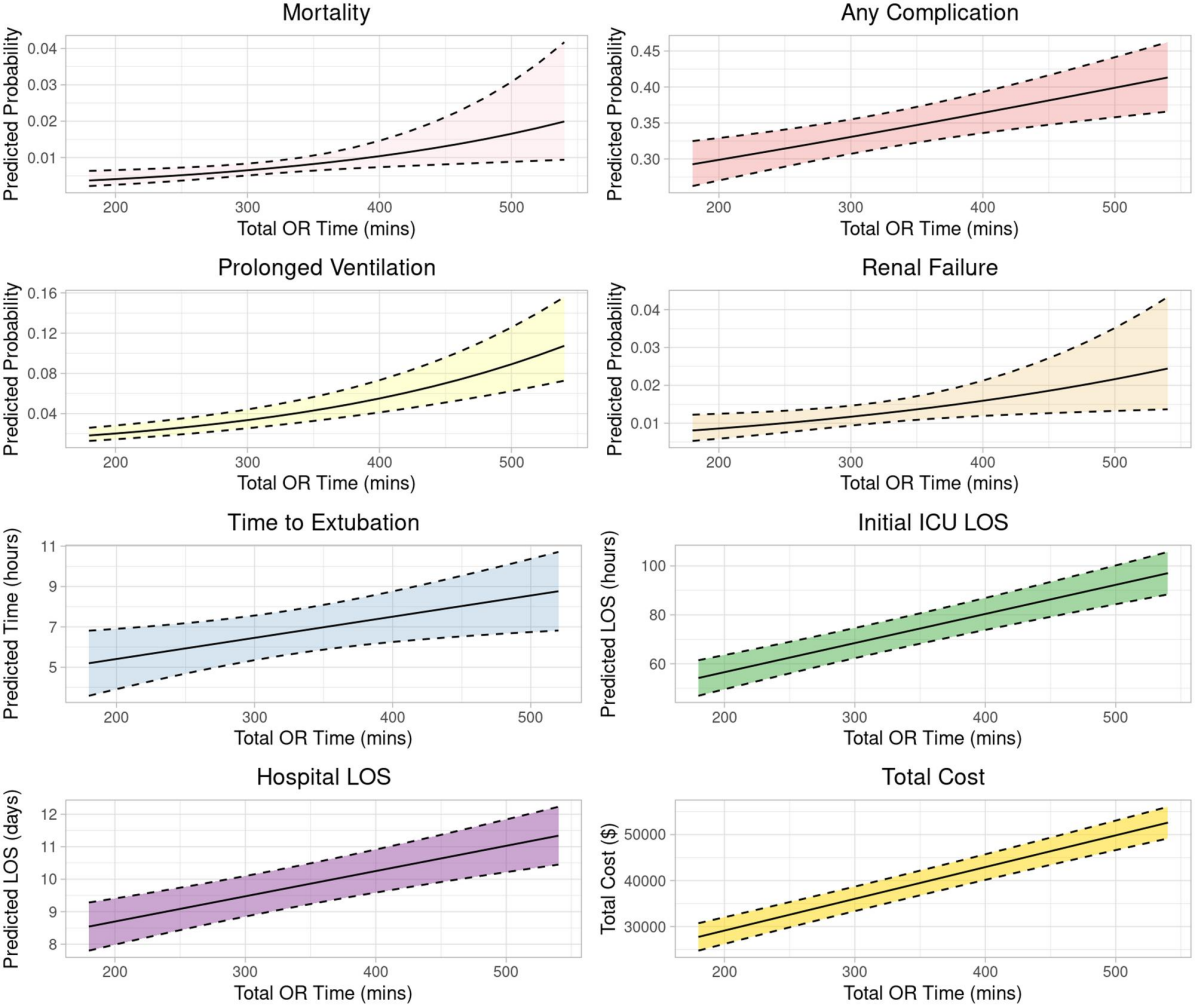
Adjusted, Statistically Significant Post-Operative Outcomes With Increasing Total OR Time.

Each additional 15 minutes in the OR was associated with a significantly increased odds of mortality (OR: 1.08, 95% CI: 1.02-1.12), all-cause morbidity (OR: 1.02 [1.02-1.03]), 0.15 hours (9 minutes) longer time to extubation (95% [0.03-0.28]), 0.07 days longer ICU LOS [0.06-0.09], 0.12 days longer hospital LOS (beta coefficient: 0.12 [0.08-0.17]) and increased total hospitalization cost by $1035 [$889-$1182] (**Table S3**). With individual post-operative complications, longer total OR time was associated with renal failure and prolonged ventilation (**Figure 2**).

### Total surgery time

**Figure 3.**
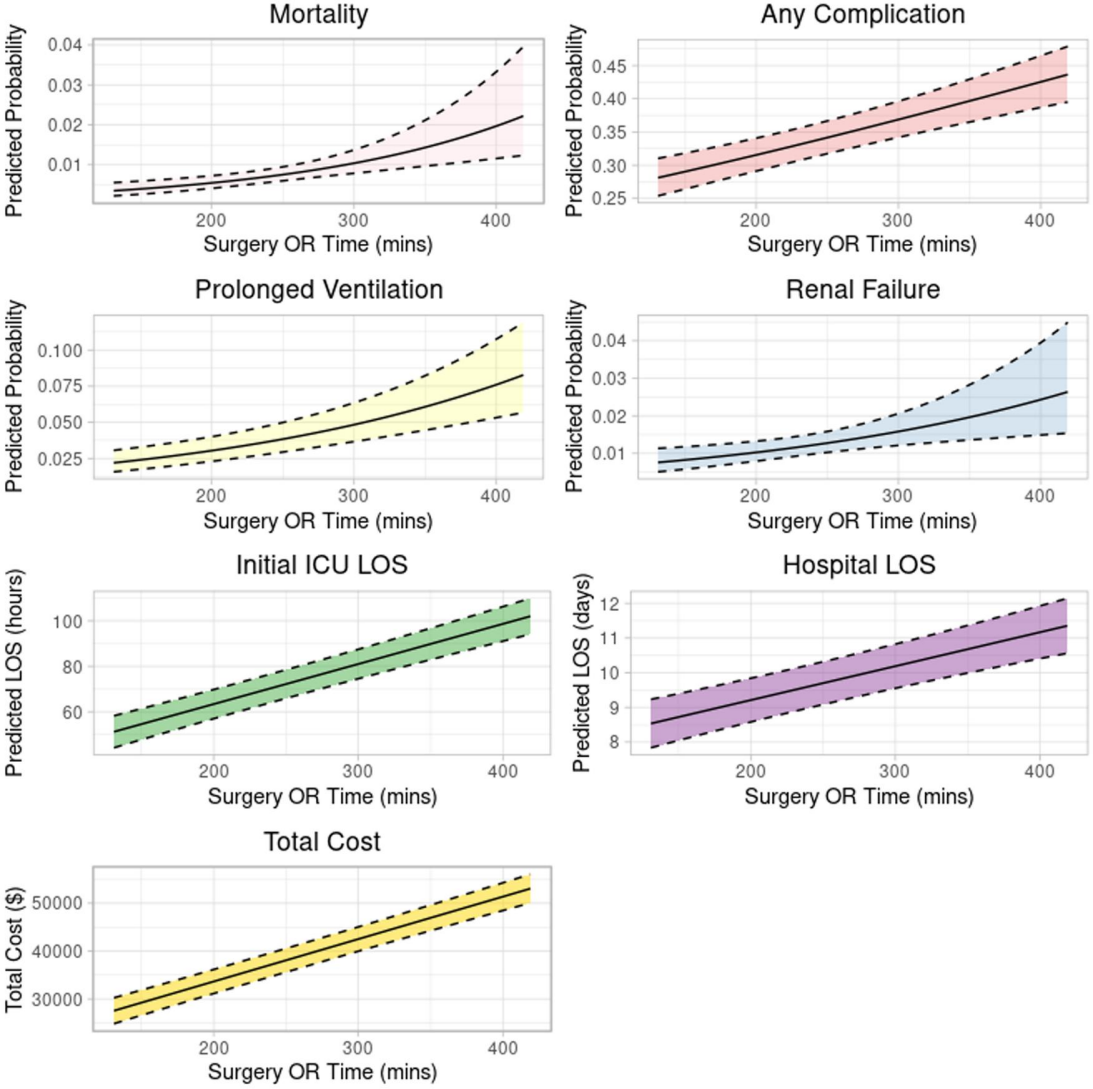
Adjusted, Statistically Significant Post-Operative Outcomes With Increasing Total Surgery Time.

Each additional 15 minutes of total surgery time was associated with a significantly increased odds of mortality (OR 1.09 [1.05-1.16]), any complication (OR 1.03 [1.02-1.04]), 0.11 days longer ICU LOS [0.09-0.13], 0.15 days longer hospital LOS [0.1-0.2], and $1329 higher total cost [$1184-$1473] (**Table S3**). With individual post-operative complications, longer surgery time was associated with renal failure and prolonged ventilation (**Figure 3**).

### Non-surgery or time

**Figure 4.**
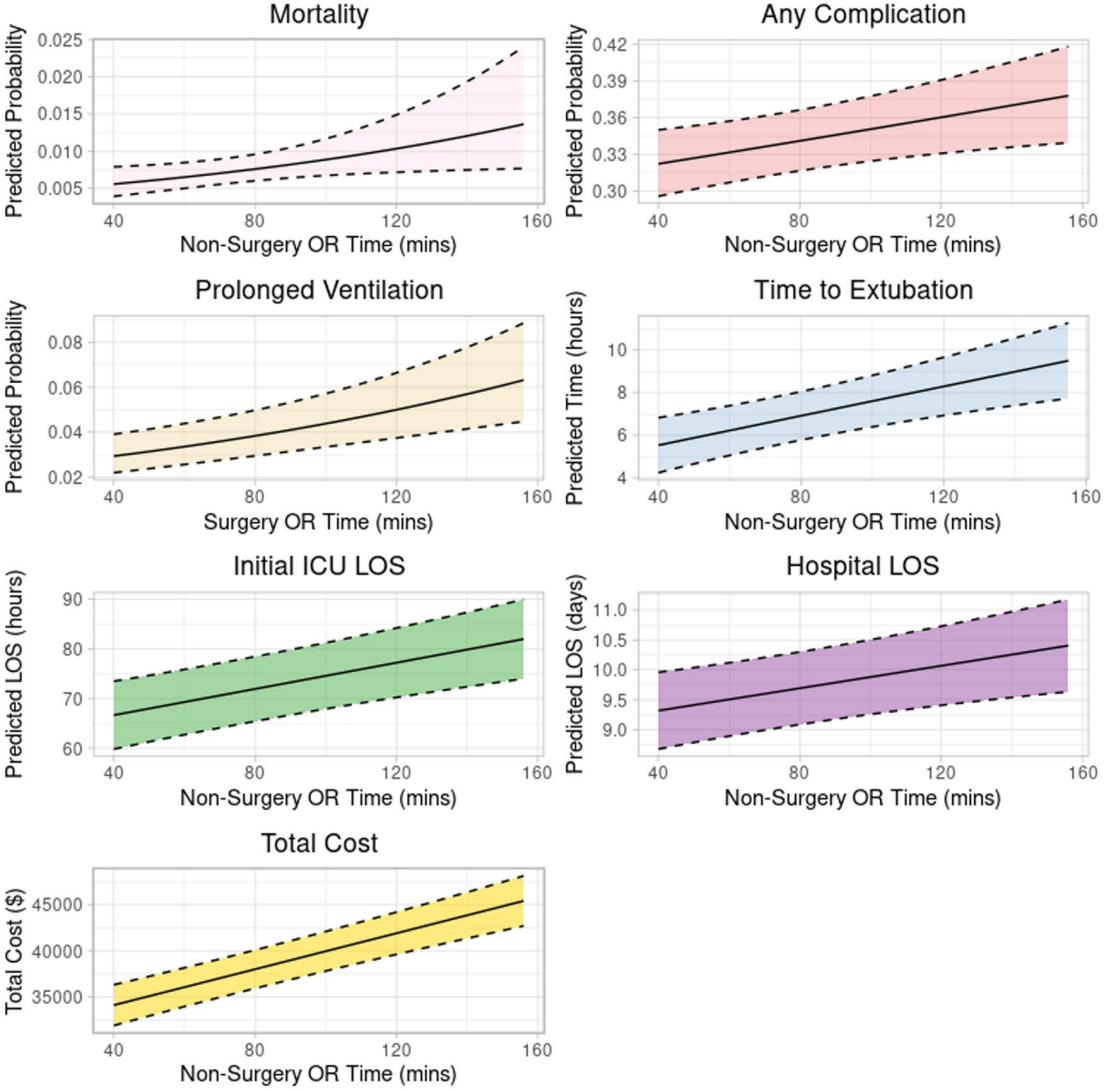
Adjusted, Statistically Significant Post-Operative Outcomes With Increasing Non-Surgery OR Time.

Each additional 15 minutes of non-surgical OR time was associated with a significantly increased odds of mortality (OR: 1.13 [1.02-1.24]), any complication (OR: 1.03 [1.02-1.04]), 0.08 days longer ICU LOS [0.05-0.12], 0.13 days longer hospital LOS [0.04-0.23], and $1463 higher total cost [$1138-$1787] (**Table S3**). With individual post-operative complications, longer non-surgery OR time was associated with prolonged ventilation (**Figure 4**).

### Off-CPB OR time

**Figure 5.**
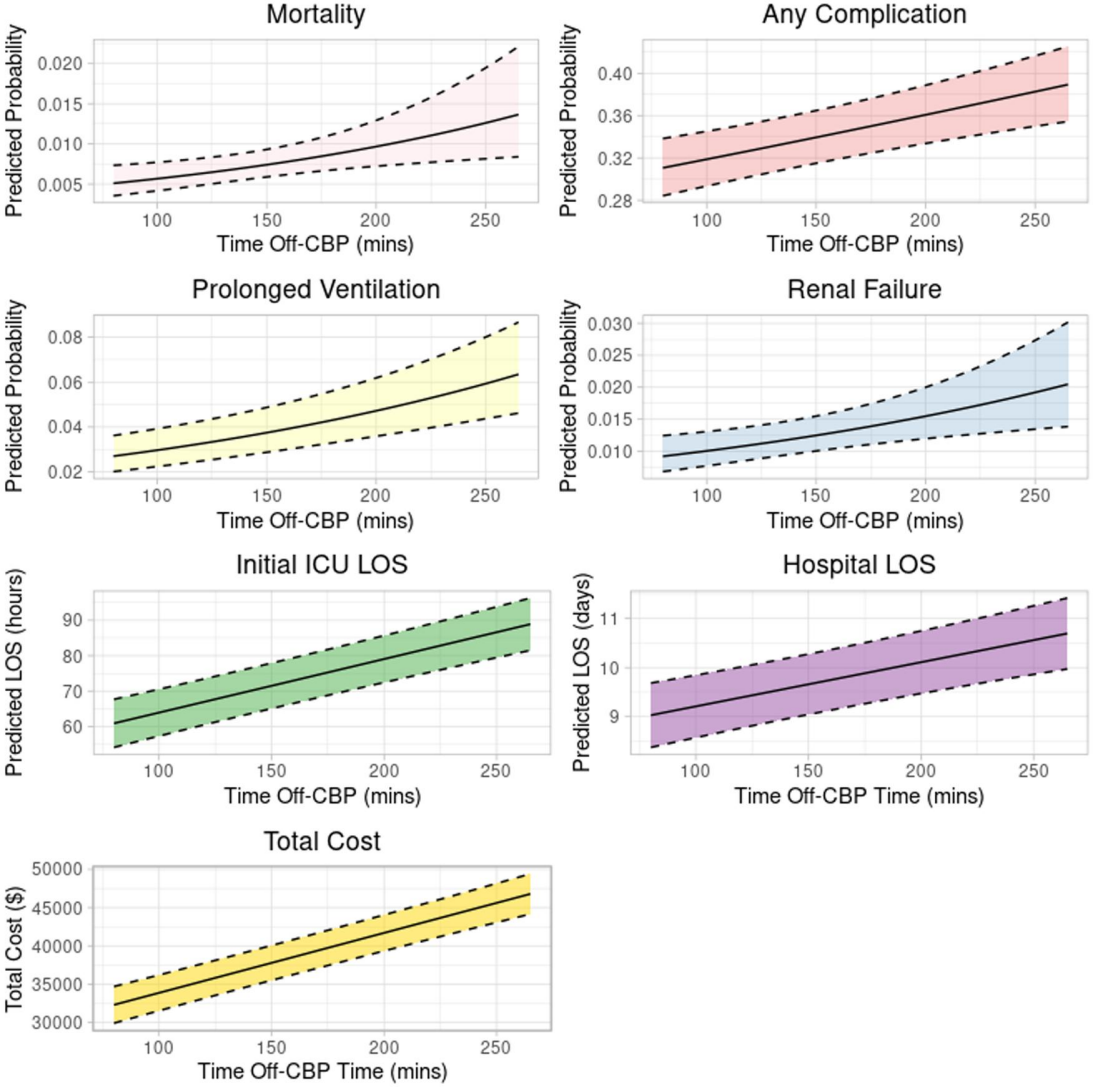
Adjusted, Statistically Significant Post-Operative Outcomes With Increasing Off-CPB Time.

Each additional 15 minutes of off-CPB OR time was associated with a significantly increased odds of mortality (OR: 1.08 [1.03-1.14]), any complication (OR: 1.03 [1.02-1.05]), 0.09 days longer ICU LOS [0.08-0.11], 0.13 days longer hospital LOS [0.09-0.18], and $1175 higher total cost [$1009-$1341] (**Table S3**). With individual post-operative complications, longer off-CPB OR time was associated with prolonged ventilation and renal failure (**Figure 5**).

### CPB time

**Figure 6.**
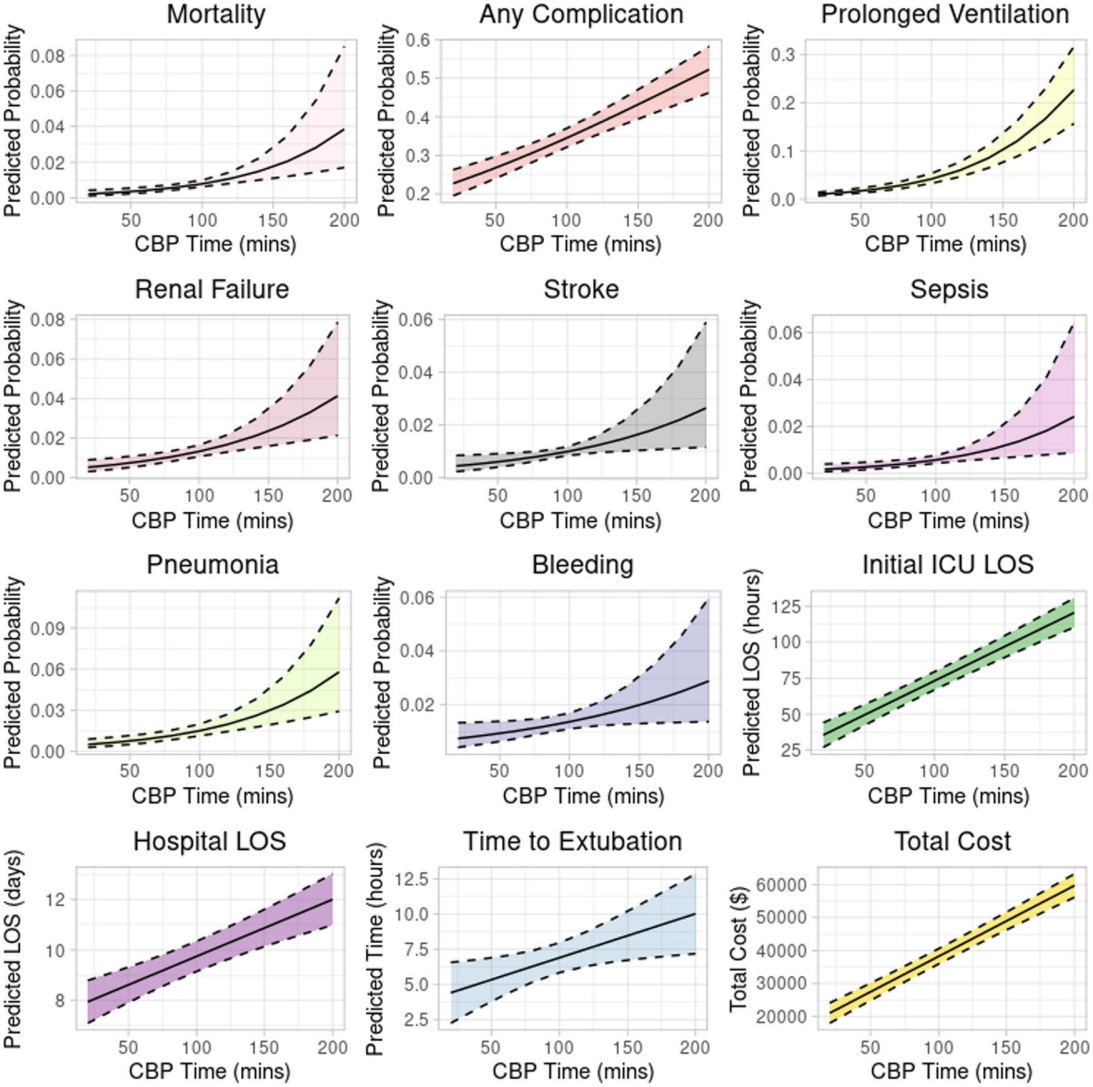
Adjusted, Statistically Significant Post-Operative Outcomes With Increasing CPB Time.

**Figure 7.**
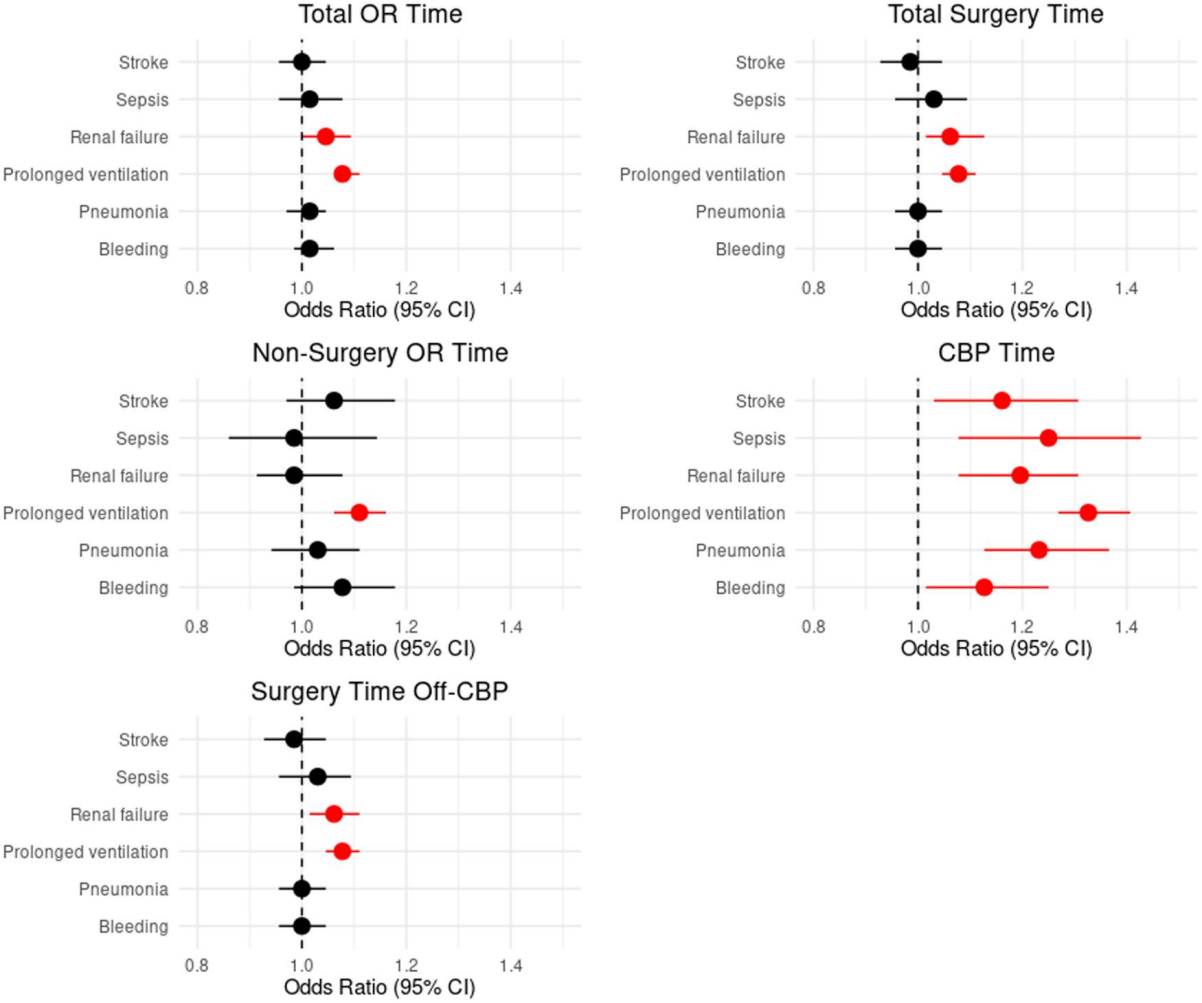
Impact of Additional 15 minutes Spent in the OR on Complications.

Each additional 15 minutes of CPB time was associated with a significantly increased odds of mortality (OR: 1.27 [1.13-1.42]), any complication (OR: 1.11 [1.08-1.13]), 0.46 hours (27 minutes) longer time to extubation [0.09-0.84], 0.30 days longer ICU LOS [0.08-0.11], 0.34 days longer hospital LOS [0.23-0.47], and $3223 higher total cost [$2830-3616] (**Table S3**). With individual post-operative complications, longer CPB time was associated with stroke, sepsis, renal failure, prolonged ventilation, pneumonia, and bleeding (**Figure 6**). A Forest plot of the odds ratios for the studied OR time points are summarized (**Figure 7**).

### Teaching status on or time

Of all the studied time points, programs with cardiothoracic fellowships versus no training programs were only associated with a significantly longer non-surgery OR time, with no significant difference in total OR time, surgery time, off CPB OR time, and CPB time.

## DISCUSSION

It is well known that prolonged CPB time in cardiac surgery patients is associated with increased morbidity and mortality. Given the overall lack of literature on the impact of non-CPB time and non-surgical time spent in the OR on outcomes in cardiac surgery patients, our goal was to study if various non-CPB time points spent in the OR have an impact on patient outcomes, as this has significant clinical relevance. We found that with each 15-minute increase in time, all of the 5 studied time points (total OR time, total surgery time, off-CPB time, non-surgery OR time, and CPB time) were associated with significantly increased mortality, morbidity, ICU LOS, hospital LOS, cost, and prolonged ventilation. Longer total surgery time, off-CPB time, and CPB time were additionally associated with significantly increased renal failure. CPB time alone was additionally associated with significantly increased time to extubation, stroke, sepsis, pneumonia, and bleeding. The teaching status of the hospital is significantly associated with a longer non-surgery OR time, with no significant difference in other time points. In our data, there was a wide variation of OR times seen and there was a weak correlation between surgical and non-surgical time.

Longer total OR time and total surgery time were associated with worse patient outcomes, LOS, cost, and complications. This has been demonstrated across multiple surgical disciplines as shown in a 2018 meta-analysis by Cheng et al showing that increased total operative time and increasing time increments significantly increase the odds of complications across multiple non-cardiac surgical specialties.^8^ Studies performed in cardiac surgery patients are more limited. In a 2008 study of 337 CABG patients, studying time from incision to patient leaving the operating room, Chu et al found that increased time significantly increased ICU LOS and ventilator time in a univariate analysis and significantly increased ICU LOS only in a multivariate analysis, with no significant difference in hospital LOS.^10^ They also found that every 30-minute increase in time was associated with 4.3 more hours of ICU stay.^10^ The findings of increased ICU LOS are similar to that of our study, however the time point used in the study by Chu et al is a combination of the total surgery time and post-surgery OR time. As both total OR time and total surgery time include CPB time, it is not surprising that an increase in these time points leads to worse patient outcomes.

Longer non-surgery time and its impact on CABG patient outcome have not been studied in the literature, and is 1 of 2 time points studied in our article that do not include CPB time, and therefore is not subject to the known influence of prolonged CPB time on patient outcome. We found that increases in non-surgery time are associated with significantly increased mortality, morbidity, ICU and hospital LOS, cost, and prolonged ventilation. Somlo et al showed that in cardiac surgery patients, delayed transfer from the OR to the ICU, a time point which equates to a fraction of the non-surgical OR time and total OR time used in our study, is associated with significantly longer ICU LOS and blood loss, with increased 30-day readmission, 30-day mortality, and cost,^11^ which are similar to the findings in our study. There are multiple contributing factors that may lead to increased surgery and non-surgery time, including the lack of standardized protocols, lack of close loop communication with anaesthesia, surgery and nursing teams, lack of awareness that non-bypass and non-surgery time can have a negative impact on patient outcomes, and the shortage of adequate health care and nursing personnel. Additionally, system resources can have an impact on the time spent in the OR as well. Inadequate operative room staffing and lack of immediate help to get the appropriate instruments and supplies, variability in anaesthesia practices and experience, as well as the lack of ICU resources to take the patient as soon as the surgery is done, can prolong OR time. Somlo et al proposed that standardization of OR practice, a dynamic work design, fostering an environment of discussion, and the integration of software-based analytics can contribute to improved OR efficiencies.^11^ Additionally, standardization of anaesthesia and nursing workflow through utilization of checklists and handover protocols can improve efficiency in the OR. Each potential variable contributing to pre-incision and post-surgical time and delays can be targeted to improve efficiency. Assessing the OR as a system and determining opportunities to increase efficiency of the team to limit non-surgical time may be beneficial to post-operative outcomes in CABG patients. Further studies and system-based analyses need to be performed to determine the specific variables that can be targeted to improve OR efficiency.

Similarly to the outcomes seen with non-surgery time, longer off-CPB time was associated with significantly increased mortality, morbidity, ICU and hospital LOS, cost, prolonged ventilation, and renal failure.

The findings in our study are consistent with the expansive literature showing that longer CPB time is associated with worse outcomes. All of the morbidity, mortality, LOS, and cost outcomes we studied were significantly worse with longer CPB time. The literature suggests that a CPB time of greater than 3-4 h should be used as a cut-off as times greater than this are associated with poor outcomes,^1^^,^^3^ which is explained by the cumulative physiologic derangement that occurs through the non-physiologic conditions associated with CPB.

We found that institutions with cardiothoracic surgery training programs are associated with significantly increased non-surgery OR time, but not the other studied time points. By association, teaching status may then be associated with mortality, any complication, ICU LOS, hospital LOS, cost, and prolonged ventilation. This finding may be related to the presence of learners, however, the lack of a cardiothoracic surgery training program does not imply the lack of other surgical and non-surgical learners in the OR. The literature on the effect of teaching hospital status on cardiac surgery patient outcomes is mixed. For example, Maldonado-Canon et al showed lower 30-day mortality of valve replacement patients in teaching hospitals, and LaPar et al showed cardiac surgery teaching hospital status is associated with increased mortality and complications but is not associated with time of year.^12^^,^^13^ Further studies are required to determine association of outcomes in cardiac surgical patients in teaching hospitals.

Lastly, each additional 15 minutes of increase in any of the studied time points significantly increases cost. As more intraoperative resources are used across all involved disciplines, such as surgical (ie, suture, special instruments, opening of unused suture and instruments), anaesthesia (ie, line attempts, use of pulmonary artery catheter), perfusion, and nursing (ie, delays in handoff, incorrect count protocols), cost can compound with more time spent in the OR. Additionally, post-operative resources such as duration of ventilator use, ICU LOS, medication use, and blood product use can also impact cost. This finding has been demonstrated across multiple surgical subspecialities. In cardiac surgery patients, Somlo et al showed increased cost with delays in moving patients from OR to ICU.^11^

There are several limitations of this study: (1) The retrospective design shows clear correlation, however, since these are associations from a risk-adjusted model, they are not necessarily causal, and residual confounding may persist; (2) this study accounted for hospital-level variations by adjusting for multiple variables, however not all of the potential hospital-level variations, such as individual institutional and provider-level practice (ie, anaesthesia setup, pulmonary artery catheter use, surgical setup, usage of total arterial vs venous grafts, transfusion protocol, extubation protocols, etc) and resource availability between hospitals, can be accounted for, which contributes to a degree of bias within the data; (3) the sample size, although large, exhibits a degree of selection bias as all patients included are derived from Virginia hospitals; (4) the data cannot discriminate the aetiology of contributors to surgical and non-surgical OR time, such as staffing levels, bed holds, anaesthesia complications, bleeding complications following bypass, or intraoperative monitoring for bleeding that occurred during non-surgical time; (5) longer ICU LOS does not always correlate to medical necessity of being in the ICU as hospitals may be at maximal capacity and time to availability of a stepdown bed can contribute to longer ICU LOS; (6) using OR time is a good surrogate for OR efficiency, however this is prone to bias and adding other surrogates for OR efficiency such as supply use could decrease bias; (7) the findings in this study are relevant to isolated primary CABG, and may not be generalizable to all cardiac surgical patients in terms of patient characteristics or surgery performed.

However, we expect these findings to persist in other cardiac surgery populations and further studies particularly aimed towards identifying and controlling for confounders can be performed to better elucidate the impact on increased OR times on efficiencies in cardiac surgery.

## CONCLUSION

In patients undergoing CABG, longer OR times, both surgical and non-surgical time, and both CPB and non-CPB times, negatively impact post-operative patient morbidity, mortality, complications, and cost of care. There is a weak correlation between longer surgical times with non-surgical times. Importantly, our study demonstrates the importance of reducing non-surgery time on outcomes in CABG patients. Improving OR efficiency will result in better patient outcomes.

## Supplementary Material

ivaf304_Supplementary_Data

## Data Availability

The data underlying this article are available in the article and in its online supplementary material.
