# Supplementary material for: Impact of Operating Room Efficiencies on Patient Outcomes Following Primary Coronary Artery Bypass Surgery
Source: Interdiscip Cardiovasc Thorac Surg. 2025 Dec 18;41(1):ivaf304. doi: 10.1093/icvts/ivaf304 (PMC12774465; doi:10.1093/icvts/ivaf304)
Supplement: ivaf304_Supplementary_Data [file ivaf304_supplementary_data.docx]

**S1**

**Characteristics of Study Population**

|  | **All patients** **(N = 29,206)** |
| --- | --- |
| **Preoperative characteristics** |  |
| Age, mean (SD) | 64.77 (9.98) |
| Sex, male | 22192 (76.0%) |
| Race |  |
| White | 22934 (79.7%) |
| Black | 4075 (14.2%) |
| AAPI | 1261 (4.4%) |
| Other | 502 (1.7%) |
| Hispanic ethnicity | 20285 (97.4%) |
| BMI, mean (SD) | 30.2 (10.2) |
| Diabetes | 14447 (49.5%) |
| On dialysis | 849 (2.9%) |
| Hypertension | 26073 (89.3%) |
| Current smoker | 4884 (22.2%) |
| Alcohol use |  |
| None | 11234 (38.5%) |
| < 1 drink/week | 11407 (39.1%) |
| 2-7 drinks/week | 3794 (13.0%) |
| > 8 drinks/week | 2394 (8.2%) |
| Cerebrovascular disease | 6127 (21.0%) |
| Peripheral vascular disease | 3899 (13.3%) |
| Chronic lung disease | 8258 (30.6%) |
| Arrythmias | 1971 (9.0%) |
| Previous MI | 15922 (54.5%) |
| Heart failure (%) | 4167 (28.1%) |
| STS PROMM score, mean (SD) | 0.12 (0.10) |
| **Intraoperative characteristics** |  |
| Number of diseased vessels |  |
| 1 | 4672 (16%) |
| 2 | 11682 (40%) |
| 3 | 8761 (30%) |
| >4 | 3213 (11%%) |
| Cross-clamp time, mins, mean (SD) | 70.7 (24.3%) |
| **Postoperative characteristics** |  |
| Time to extubation, hours, mean (SD) | 12.9 (59.3%) |
| Mortality | 282 (1%) |
| Any complication | 9883 (33.8%) |
| Sepsis | 197 (0.7%) |
| Stroke | 320 (1.1%) |
| Prolonged ventilation | 1673 (5.7%) |
| Renal failure | 537 (1.8%) |
| Bleeding | 439 (1.5%) |
| Pneumonia | 512 (1.8%) |
| ICU LOS, days, mean (SD) | 2.8 (3.5) |
| Hospital LOS, days, mean (SD) | 9.8 (8.6) |
| Discharge location |  |
| Home | 23878 (82.4%) |
| Rehabilitation/care facilities | 4841 (16.7%) |
| Other | 261 (0.8%) |

Abbreviations: SD: standard deviation, AAPI: Asian American and Pacific Islander, BMI: body mass index, MI: myocardial infarction, STS PROMM: Society of Thoracic Surgeons’ preoperative risk of morbidity and mortality score, ICU: intensive care unit, LOS: length of stay **S2**

**Distribution of time spent in the OR (in mins)**

| **Percentile** | **OR Time** | **Total Surgery Time** | **Non-Surgery OR Time** | **Surgery Time Off-CBP** |
| --- | --- | --- | --- | --- |
| Min | 197 | 131 | 40 | 80 |
| 1% | 209 | 146 | 43 | 88 |
| 25% | 271 | 201 | 61 | 121.00 |
| 50% | 308 | 235 | 72 | 141.00 |
| 75% | 351 | 274 | 85 | 165.00 |
| 99% | 468 | 376 | 132 | 241.85 |
| Max | 525 | 419 | 156 | 265.00 |

Abbreviations: OR: odds ratios; CBP: cardiopulmonary bypass.

**S3**

**Summary of Outcomes for Each Additional 15 Minutes Spent in the OR**

|  | **Logistic Regression (OR [95% CI])** | | **Linear Regression (Beta coefficient [95% CI])** | | | |
| --- | --- | --- | --- | --- | --- | --- |
|  | **Mortality** | **Any complication** | **Time to extubation (hours)** | **ICU LOS**  **(days)** | **Hospital LOS (days)** | **Total cost**  **($)** |
| **Total OR time** | **1.08 [1.02-1.12]*** | **1.02 [1.02-1.03]*** | **0.15 [0.03-0.28]*** | **0.07 [0.06–0.09]*** | **0.12 [0.08-0.17]*** | **1035 [889-1182]*** |
| **Surgery time** | **1.09 [1.05-1.16]*** | **1.03 [1.02-1.04]*** | 0.12 [-0.02-0.24] | **0.11 [0.09–0.13]*** | **0.15 [0.10-0.20]*** | **1329 [1184-1473]*** |
| **Non-Surgery OR time** | **1.13 [1.02-1.24]*** | **1.03 [1.02-1.06]*** | **0.51 [0.26-0.78]*** | **0.08 [0.05–0.12]*** | **0.13 [0.04-0.23]*** | **1463 [1138-1787]*** |
| **Surgery time off-CBP** | **1.08 [1.03-1.14]*** | **1.03 [1.02-1.05]*** | 0.08 [-0.06-0.21] | **0.09 [0.08–0.11]*** | **0.13 [0.09-0.18]*** | **1175 [1009-1341]*** |
| **CBP time** | **1.27 [1.13-1.42]*** | **1.11 [1.08-1.13]*** | **0.46 [0.09-0.84]*** | **0.30 [0.25–0.34]*** | **0.34 [0.23-0.47]*** | **3223 [2830-3616]*** |

Abbreviations: OR: odds ratios; CI: confidence interval; ICU: intensive care unit; LOS: length of stay; CBP: cardiopulmonary bypass.
